# Virtual User in the IoT: Definition, Technologies and Experiments †

**DOI:** 10.3390/s19204489

**Published:** 2019-10-16

**Authors:** Roberto Girau, Raimondo Cossu, Massimo Farina, Virginia Pilloni, Luigi Atzori

**Affiliations:** DIEE, University of Cagliari and National Inter-University Consortium for Telecommunications (CNIT), Research Unit of Cagliari, 09123 Cagliari, Italy; rai.cossu@gmail.com (R.C.); m.farina@diee.unica.it (M.F.); l.atzori@diee.unica.it (L.A.)

**Keywords:** virtual user, virtual object, edge computing, QoE

## Abstract

Virtualization technologies are characterizing major advancements in the Internet of Things (IoT) arena, as they allow for achieving a cyber-physical world where everything can be found, activated, probed, interconnected, and updated at both the virtual and the physical levels. We believe these technologies should apply to human users other than things, bringing us the concept of the Virtual User (VU). This should represent the virtual counterpart of the IoT users with the ultimate goal of: (i) avoiding the user from having the burden of following the tedious processes of setting, configuring and updating IoT services the user is involved in; (ii) acting on behalf of the user when basic operations are required; (iii) exploiting to the best of its ability the IoT potentialities, always taking always account the user profile and interests. Accordingly, the VU is a complex representation of the user and acts as a proxy in between the virtual objects and IoT services and application; to this, it includes the following major functionalities: user profiling, authorization management, quality of experience modeling and management, social networking and context management. In this respect, the major contributions of this paper are to: provide the definition of VU, present the major functionalities, discuss the legal issues related to its introduction, provide some implementation details, and analyze key performance aspects in terms of the capability of the VU to correctly identify the user profile and context.

## 1. Introduction

The evolution of the Internet of Things (IoT) and the pervasive diffusion of smart objects is enhancing humans’ ability to examine reality. Huge amounts of data collected by ubiquitously deployed sensors are processed to obtain information that is used to raise knowledge and awareness of the surrounding real-world environment and achieve a wider perspective of it. Accordingly, decisions and consequent actions are going to be more rational, and this is going to generate an overall improvement in users’ lifestyles.

Technological and social advances encouraged the transition from Web 2.0 [2] to the pervasive, semantic and ubiquitous Web 3.0 [3]. Analogously, the same transition is going to affect the IoT and the role of objects within user-to-object and object-to-object interactions. By communicating with their peers and exploiting more intelligent technologies, such as cloud computing, even the most elementary sensors acquire the ability to be aware of their surrounding ambient and make complex decisions based on their context.

The impact of ubiquitous devices is envisioned to revolutionize the environments as people are used to imagining them; indeed, it will improve the management of resources such as computing and storage capabilities, and enhance the intelligence thanks to distributed computing. Accordingly, traditional environments, such as homes, hospitals and factories, just to cite a few, are becoming smart as a consequence of being able to make autonomous decisions and simplify common life tasks. Nevertheless, these new scenarios have a major drawback: objects and applications still need to be set up, managed and updated by humans, using the most various and ever-changing interfaces. Furthermore, privacy and security issues require frequent intervention from users. In addition, applications are still selected and installed manually by users, which need to browse among an exponentially increasing number of used and available ones. Therefore, if, on the one side, the mentioned technologies are unburdening humans from repetitive and tedious tasks, on the other side, these are introducing new management-related tasks that risk vanishing all relevant benefits.

Based on these considerations, in this paper, the concept of Virtual User (VU) is introduced as a component to address the challenges introduced by the previously mentioned scenarios. The VU is intended as the virtualized user, i.e., an agent deployed in the cloud and edge computing infrastructure, which provides the following major functionalities: it provides the user with a unified interface; it is able to manage the user’s context; it creates the user profile; it manages the user credentials; it manages the user’s Quality of Experience (QoE) requirements; and it manages the available computing and storage resources based on the required ones.

Accordingly, the contributions of the paper are the following:

- We provide a first comprehensive definition of Virtual User and we describe the functionalities that it has to implement to take a leading role in the implementation of major IoT cloud-based infrastructures and applications;
- We provide a description of the architectural components of the Virtual User and we provide some details of the implementation that have been carried out to develop a prototype;
- We discuss the important legal issues of user privacy and manipulation of the data that are acquired about the user and how the threads behind the introduction of these components are handled;
- We present some experimental results for the evaluation of the capability of the proposed solution to identify correctly the profile and context of the Virtual User.

After briefly analyzing the reference past works in Section 2, we provide the definition and present the needed functionalities in Section 3. In Section 4, we illustrate the VU components and provide major implementation details. In Section 5, we discuss the legal implications related to the introduction of the VU. In Section 6, we present some experimental results. Conclusions are finally given in Section 7.

## 2. Past Works

User profiling has been a research topic for many years now and is the basis of the targeted advertising systems that we find in all websites. It is mainly based on well-known pattern recognition methods that in many cases led to the registration of many patents. In the IoT field, we talk about user profiling in terms of context awareness or cognitive IoT. The problem with these systems is that they are focused on the specific use case or the particular application and all user information is saved and analyzed separately, resulting in duplicating learning processes or losing important user characteristics transversal to different applications. In the following, we present an analysis of the state of the art on user profiling, with also a particular focus on IoT scenarios. In this way, we want to highlight what is already present and we can reuse and what is still lacking in the context of the IoT.

### 2.1. User Profiling and Mobile Agents

The concept of VU has often been used in the past to assess the level of accessibility of the system in the different stages of product development. In [4], the authors’ goal is to introduce tests based on simulation and virtual reality in the automotive, intelligent living spaces, workplaces, infotainment and personal health care applications. A framework has been defined and developed with the aim of ensuring that future products and services are systematically designed for all people, including those with functional limitations. An open simulation platform consisting of several tools provides automatic simulation feedback on compliance with design guidelines/methodologies and service quality requirements. To this, physical, cognitive and detailed VU models were integrated to support simulation and testing at all stages of product planning and development.

The work described in [4] is the closest definition of VU to the one we provide in this article. However, there are significant differences as we focus on implementing and managing the IoT platform and applications, making our work remarkably innovative. Nevertheless, in order to achieve relevant objectives, we use some key technologies already used in other contexts that we will briefly examine below. These are mainly related to the generation and management of the *user profile and context*, as well as the adoption of *cloud computing* and *virtualization technologies*.

User profiling techniques are deeply investigated in [5] defining the three main steps: acquisition; learning and representation; adaptation. These tasks are mainly executed through agents, which, as personalized user assistants, must have knowledge about the user and the related application domain. Indeed, the available information about users and agents’ ability to represent user interests are the key factors to successful virtualization of the user. The user-programming approach requires agents to be programmed by users by means of scripts or rules for processing information related to a particular task. The knowledge-engineering approach requires an agent to build a domain-specific knowledge of both the application and the user. Since it is highly domain specific, agent knowledge is consequently hardly adaptable to different application domains [6]. However, agents can be endowed with machine-learning mechanisms to allow them to acquire the needed knowledge. All these technologies are key for the realization of our VU, where we have to understand specific requirements to tailor existing solutions to our specific case.

In such cases, the agent is provided with limited background knowledge, and it learns the behavior from the user and from other agents. Users can train agents by means of hypothetical examples of events and situations and providing their behavior in those cases. This is the case of the programming by example (PBE) paradigm [7]. The profiles can be modeled with the knowledge implicitly or explicitly acquired from users, so as to have a division between explicit and implicit user profiles. An explicit user profile is created by means of a set of questions designed to acquire user interests and preferences [8]. A simple model of user preferences and interaction patterns can be obtained by continuously observing user actions. Implicit knowledge acquisition is usually preferred due to its little or zero impact on the user regular activities. The observation time needed by the agent to learn user behavior is a critical element. To reduce this time, agents can use the knowledge shared by other agents. A number of methods have been developed for collaborative or social filtering of information [9]. The user profiling by stereotypes approach is based on the assessing of generalizations about communities of users [10]. The use of stereotypes for user-profile acquisition has been shown to be useful in those cases where a fast, but not necessarily accurate, evaluation of user interests is required. The adaptation ability of the agents requires learning mechanisms, usually divided into supervised and unsupervised learning methods. The former requires a pre-classified set of training examples, in which each training example is assigned a unique label indicating the class it belongs to among a finite set of possible classes. Conversely, unsupervised learning methods do not require pre-classification of the training examples; these algorithms aggregate examples which share common characteristics in clusters. In the user profile, there are pieces of information that are persistent and others that change over time [11]. Therefore, the adaptation of user profiles is an essential requirement. To this, imitating the gradual process of natural forgetting, several forgetting mechanisms have been proposed in the literature to adapt user profiles [12].

Other than the user profile, the VU should continuously consider the context. Indeed, detecting, describing, and using the context in IoT applications are some of the main challenges so that objects can automatically adapt accordingly. Many recent architectures proposed by the literature tackled this issue, particularly those that are based on virtualization techniques [13,14]. This capability to acquire, analyze and interpret information about the surrounding environment is the context awareness. Awareness is needed to simplify the discovery of information about the object itself when this information cannot be easily reached, is not made explicit in the required format, or needs aggregation with other information sources before it can be used [15,16]. Additionally, the awareness of the context is key for the implementation of the appropriate level of security [17]. Discovered information is represented by resources and services that are made available by other nodes, as well as data gathered by them. Strictly related to context awareness is cognitive management: the knowledge of the environment where the object is operating is used to make relevant decisions and act upon them [16]. Whenever a change in the context is perceived, information is processed through the use of optimization algorithms and predictive models [18]. According to the obtained results, the network objects autonomously reconfigure in order to adapt their behavior to changes.

### 2.2. Virtualization in Cognitive IoT

The latest introduction of cloud computing characteristics into the IoT landscape has been a significant breakthrough in deploying advanced apps, particularly for cognitive IoT frameworks. Here, significant study projects implemented the virtualization feature, as in [13].

In this project, the proposers define the Virtual Object (VO) as the virtual alter ego of any real-world object (RWO), which are dynamically created and destroyed. Cognitive technologies guarantee a constant link between RWO and VO and ensure self-management and self-configuration. A further composition of VOs results in Composite Virtual Objects (CVOs) that are super entities with inherited features from the inner VOs and other augmented capabilities. In the Lysis platform [19], the devices have virtual counterparts with socialization capabilities, named Social Virtual Objects that run in a Platform as a Service (PaaS) infrastructure and are supported by the large amount of computational and storage capabilities available in datacenters. When the latency caused by interactions among the elements of the IoT environment in the cloud is unacceptable, edge cloud allows for moving cloud services much closer to end-users by introducing intelligence and flexibility into network edge nodes able to host cloud applications [20]. This allows for manipulating user requests before crossing the network towards data centers so as to achieve better performance. This solution perfectly matches the promising Cloudlet and Fog computing paradigms [21]. According to the Fog computing paradigm, network edge devices are evolving into micro Cloud servers, able to host not only advanced network functions but also applications and user related software. This allows for instantiating distributed functionalities, accommodating the desired level of QoE (Quality of Experience) and workload/traffic volumes, closer to the end-users. Furthermore, the distributed architecture composed by large-scale geographically deployed nodes natively possesses scalability properties. To meet these challenges, in [22], authors propose adopting edge cloud technologies in the implementation of the Lysis platform. This work highlights the changes introduced in a traditional cloud-platform based implementation, consequent to the use of edge cloud technologies, which are able to detect the need for moving to the geographical location of the virtual object and to handle the intercloud mobility of related processes and data.

## 3. Definition of Virtual User and Major Functionalities

In our vision, the VU is the “*trusted digital counterpart* of the human it represents, facilitating her *integration into the technological digital world* reducing the burden of following the tedious processes of setting, configuring and updating IoT applications she is involved in *and exploiting at the best the relevant potentialities*, taking always into account her quality of life”. Accordingly, these are the major VU objectives:

- *Integration into the digital world*: the degree of involvement of people is constantly increasing with an increase in the time we dedicate to these activities. As a result, even if this technological revolution has had a positive impact on our lives so far, we are at the point where further progress could nullify the expected benefits unless we find a way to free ourselves from the related management burdens through the help of a trusted companion, i.e., the VU.
- *Exploiting to the best of its ability the IoT applications’ potentialities*: many people ignore the existence of some applications that could solve some of our daily problems. This is the case, for example, of applications that can estimate the number of people waiting at the post office or doctor, and which could help save time wasted waiting; applications that can tell in which shop we can find the cartridge for our printer that we suddenly finished. Based on our profile and activity, the VU can suggest the most appropriate applications to use (and those that should be removed) by autonomously exploring the huge and dynamic market of IoT applications.

**Figure 1:**
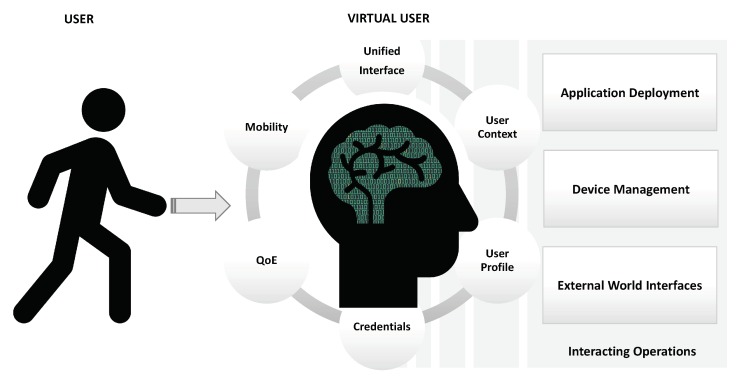
The VU scenario.

We believe that these objectives can be achieved by assigning to the VU the following functionalities (see Figure 1):

- *Unified interface*: The VU provides the user with common and unified access to the digital world that should mask the complexity of device and application management.
- *User context*: The user is involved in multiple applications, from managing smart devices at home for energy efficiency to using the car and calculating the best route to reach the scheduled appointment, from the management of unified communication tools to document sharing for work and entertainment. The knowledge of the user’s context of interest is of fundamental importance for all these applications as they should take action on behalf of the user herself, always keeping in mind the updated context.
- *User profile*: In addition to the context, the user profile provides important information about her characteristics. It is built by observing the user for longer periods of time (with respect to the context analysis) and therefore changes slowly. It has something to do with some distinctive aspects of habits, such as: preferences, interests, work, social relationships and more.
- *Credentials*: Access to our documents and information is becoming increasingly complex and difficult due to the increasing number of services we use every day on the web. Since our activities are mainly based on the use and interconnection of many services, we must continuously recover and use passwords that we are unable to remember and manage correctly.
- *Quality of Experience (QoE)*: The quality of the experience is undoubtedly a crucial concept in providing successful services and products. However, although it is easily understandable, it is complex to implement effectively in real end-to-end systems/networks. Furthermore, it is subjective, as it should follow the user’s perception, which changes from person to person. Consequently, appropriate personal models should be created and adopted for each user in order to maximize the benefits and success of the applications used.
- *Mobility*: The introduction of the edge computing paradigm fosters the idea of smart mobility between infrastructures in which to run software and applications related to the user.

## 4. The Proposed Model

**Figure 2:**
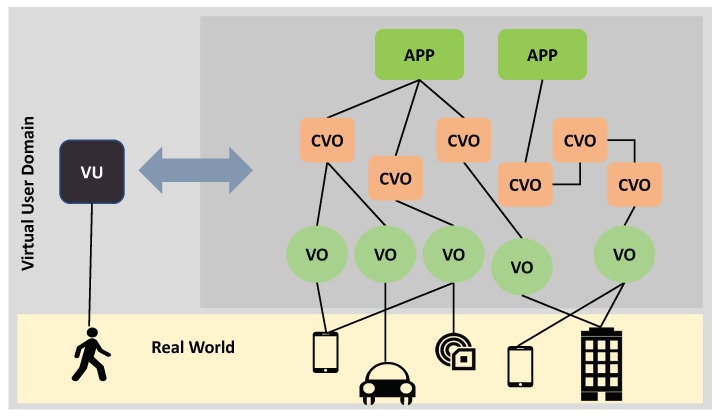
Interaction of the VU with the other major components in an IoT-platform.

As highlighted in Section 2, the previous research on IoT architectures is based on the concept of VO and CVO, which are the basis of our architecture as well. Consequently, the VU interfaces with the following levels: the Real World, VO, CVO and Application levels, as shown in Figure 2. The main concept is that the Real World Object has a virtual counterpart running in the digital world that participates in the execution of IoT applications. Similarly, the human has a virtual representation that takes on the role of a proxy between the digital world and the user.

We have developed the components needed for the realization of major VU functionalities as shown in Figure 4. These have been implemented as an extension of the Lysis platform [19]. In the following three subsections, we present the semantic description of VUs’ profile, some components’ implementation details, and an implementation overview of the VU, with particular attention to the creation of its profile.

### 4.1. Semantic Description of the VU Profile

**Figure 3:**
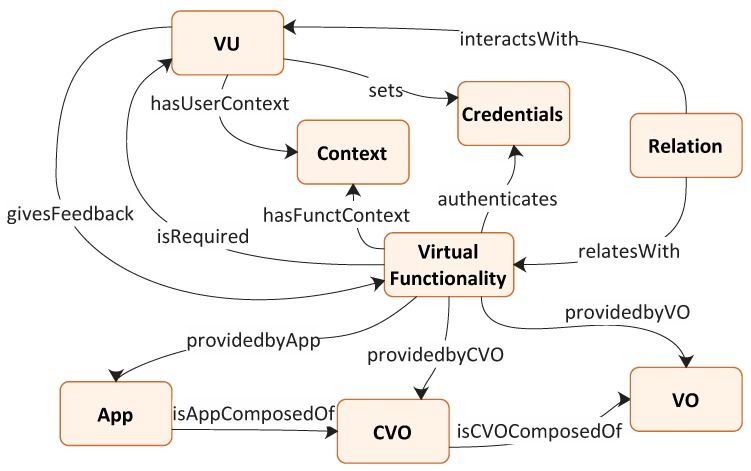
Semantic description of the VU profile [1].

One of the key elements of VU management is the abstraction of its features and functionalities. Since the VU represents the digital counterpart of a user, its model is based on the virtualization of the user’s own functions that constitute the user profile. The semantic description proposed in this document for the VU is based on the ontologies presented in [16,23]. Figure 3 depicts the main modules that are used in the proposed architecture to describe the VU profile.

*VU*: It provides a description of the main information related only to the user. It can be: requested directly to the user through the user interface (e.g., name, date of birth); taken from other architecture virtualizations using the IoT interface (e.g., the music preferences inferred from her music apps); inferred from the context by means of the context management functionality (e.g., the user status, or else if the user is walking, running, standing still, etc.).

*Virtual Functionality*: It defines some features, such as temperature monitoring, alarm system, route planning, which can be supplied by a VO, a CVO or an App. This module also describes which input parameters are required, which output parameters are supplied, and which requirements (i.e., Quality of Information, QoE) are defined for this functionality. A VU may require a feature and then it can provide feedback after it has been performed.

*VO*: It includes the characteristic parameters associated with a virtualized object, as defined by the iCore architecture’s VO information model. If it is related to an ICT object, it also includes a description of it, along with information about the type of ICT object it represents, e.g., wearable, portable, mobile.

*CVO*: It provides information that describes the CVO’s main features. It is connected to the VOs of which it is made up, as depicted in Figure 2.

*App*: It defines the app’s main parameters. It is connected to the CVOs of which it is made up, as depicted in Figure 2.

*Context*: It includes context-related information such as time and location (both coordinates and location description, e.g., outdoor, car, hospital, office). The context can either be related to the location of a user or to the location associated with a particular functionality.

*Credentials*: It defines the keys that a VU can set and can be used to authenticate a virtual functionality, be it a VO, a CVO or an App. They are used by the functionality of Identification and Authorization Management as described below.

*Relation*: It describes a relationship that connects two VUs or a VU to a Virtual functionality. If it connects two VUs, it reflects a bond of friendship between them, allowing certain functionalities that are managed by the Social Network Management functionality (see below). In the event that the relationship links a VU and a Virtual functionality, it describes a connection between the VU and the VO, CVO or App that provides the functionality, which is usually an ownership connection. The relationship between devices and users is of key importance since it characterizes both the Virtual Objects and the Virtual User. Devices belonging to a user may have different setting parameters and relevance with respect to similar devices belonging to other users. Furthermore, the trustworthiness of the information provided by an object may be affected by the relation of the VU with that object. Therefore, for instance, some private information will be only shared among objects that have an ownership relation with the VU, i.e., those that belong to the virtualized user. Likewise, relationships resulting from the presence of devices in locations frequently visited by the user, such as home, work, gym, metro, etc., can provide meaningful information about the reference context. Of course, the strength of a VU–VO relationship is also crucial, and this information then impacts user profile building and/or context evaluation.

### 4.2. Components of the Virtual User

**Figure 4:**
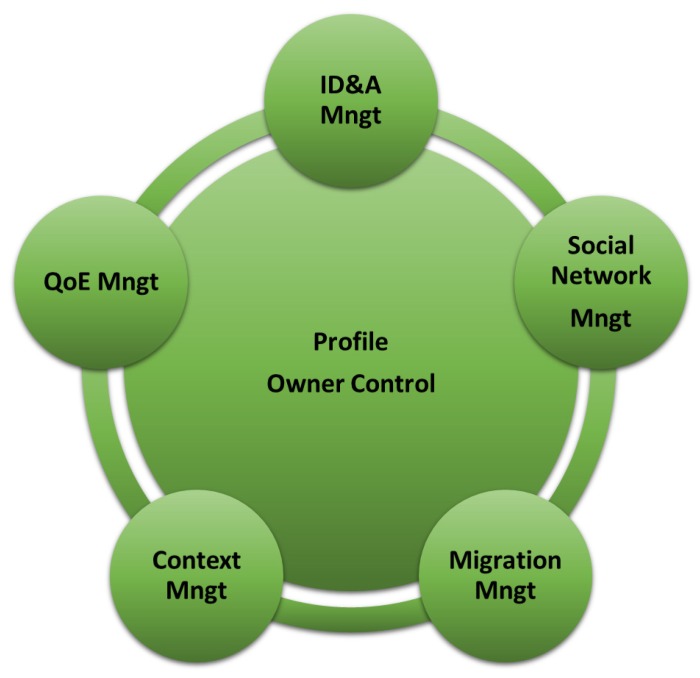
Main components of the VU [1].

Figure 4 shows the main components of the VU, which relies on two core elements:

**Profile:** As described in the previous subsection, the profile describes the user’s main features such as: preferences, interests, work activities, relationships, and others. It involves both user-provided static data and dynamic data gained through learning processes. Users are classified as far as possible according to preferences and description based stereotypes. The user knowledge modeled in the profile can be acquired from the user explicitly or implicitly. The explicit component is obtained through a set of questions designed to acquire precise interests and preferences. Since it involves a large degree of user involvement, discomfort and loss of interest in giving the responses must be minimized. By watching the user behavior to infer habits, preferences and interests, the VU makes use of pattern recognition processes in the acquisition of implicit elements. However, the gained information implicitly needs a degree of interpretation in order to comprehend the intentions of the actual user and can, therefore, be subject to evaluation mistakes.

**Owner Control:** Users have complete control over the decisions taken by VUs on their behalf and can act to give the required adjustments. Advanced users may have complete control over their VUs to manage the scripts that define their behavior, while less qualified users may cooperate to train the VUs from time to time. This module is crucial in the early learning stage in evaluating the user’s choice regarding the safety and privacy levels of all system records.

The additional functional components of the VU are the following:

**ID and Authorization (ID&A) Management:** This component is essential to the management of the access credentials for all user’s resources. In traditional systems, the user generates access credentials for her devices in the management platform and, in some cases, can give credentials to other users to enable access to them. The VU performs this task on her behalf on the basis of past decisions and preferences learned. For example, an IoT service requires an access key, which is generated by the VU for that service while notifying its VOs of potential requests. A time-to-live key can be activated by the VU to force the service to request a fresh key. It can also decide that, by assigning a budget, the key enables restricted resource consumption so it turns off once the resource budget is finished. This credential creation and destruction method improves the general system security and significantly decreases user effort, particularly in various IoT services’ instances. Indeed, repeated access requests make the customer accept the applications without even examining them. This module also uses heuristics to detect unusual behaviors when using credentials and may decide to delete them if it detects behaviors that are not suitable to the user’s level of security and privacy.

**Quality of Experience (QoE) Management:** This module is intended to collect all user behavior data in different situations in order to customize the QoE models for each user-interested application. Usually, this is achieved by choosing weighting parameters that amplify the significance of a particular application aspect to others. For example, some users are more interested in aspects of security than in aspects of aesthetics. The models therefore dynamically drive the way certain functionalities are implemented in different layers, ranging from the representation and exchange of VO information to the presentation layer that directly affects the perception of application consumption.

**Social Network (SN) Management:** Like people who set up friendships with other peers to exchange data or engage in cooperative activities, VUs also establish relationships with other VUs. They are obtained from the owner’s human social networks interactions (such as from Facebook and Twitter activity) and by monitoring the behavior of users (such as other persons being met). This element allows the VU to orchestrate IoT services that involve many people’s preferences, enhance the learning process in defining user stereotypes and the general reliability of data exchanged through the distinct levels of connection in social relationships.

**Context Management:** Context awareness is essential to enable the VU to understand which resources and functionalities it has at its disposal and which are best suited for executing apps. The element of context management involves context learning and cognitive management. Context learning allows the VU to gather data about the context in which it is placed/moving and to gain understanding about the VOs and VUs that can communicate with it. To this, the context learning functionality takes advantage of the concept of Descriptive Numeric Array (DNA), which is an array of data consisting of the elements of the semantic description of the VU profile that specifically identify it (e.g., the values of its characteristic parameters, its functionalities). Cognitive management includes all those processes that allow the use of context-related data to make choices about the best VOs and VUs that can contribute to the execution of the application. For example, if the VU requires the temperature value inside a room where it is located, the most suitable VUs (and related VOs) to answer are those located in the same room, i.e., those with a DNA whose location-related elements are similar to the VU’s DNA which made the request. Cognitive management is also accountable for optimizing the use of resources and managing faults.

**Migration Management:** The VU has the capacity to decide in which PaaS infrastructure to operate, which is the standard main cloud or the edge cloud most of the time. The VU may involve a migration to operate closer to the customer depending on the required service and associated latency conditions. For this purpose, the VU takes the context data, user preferences and practices into account and provides a forecast of the future requirements of the user to optimize the general quality of service perceived by the user.

### 4.3. Implementation Overview

**Figure 5:**
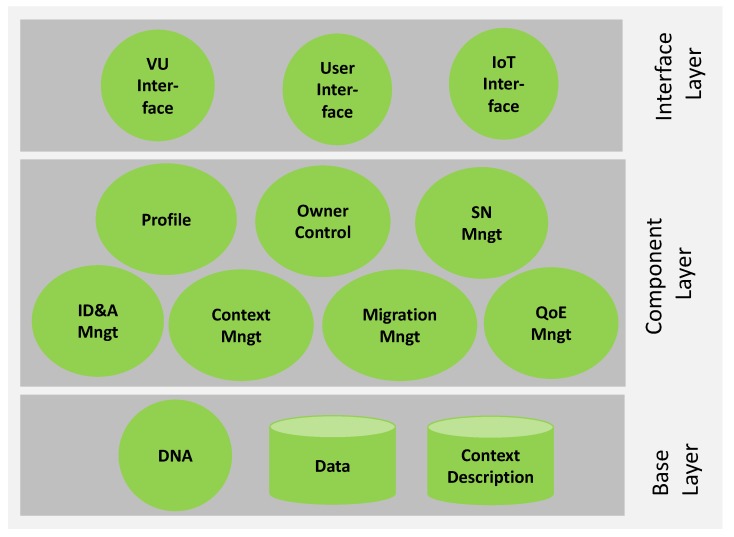
The functional layers of the VU [1].

We adopted a scheme made up of three major layers as shown in Figure 5 to integrate the VU in an IoT-cloud system. The base layer involves the database for storing and managing information, context descriptors and DNA of the user, which contains characteristics that define all user information. The component layer includes the tools for implementing the functionalities described in the previous subsection: profiling, owner control, ID and authorization management, management of human social networks, context management, and management of migration. The upper layer encompasses all the interfaces with the other VUs, IoT components (VOs, CVOs and Apps) and people. Recall that a VU’s interaction with the other IoT-cloud platform’s major parts is shown in Figure 2.

The creation and update of VU profiles according to the context and relationships established with other VUs and VOs relies on users’ monitoring performed by ICT objects. Indeed, users must be observed by ICT objects that act as transducers and convert physical features into digital descriptors in order for them to exist in the virtual dimension. Nowadays, individuals are constantly surrounded by electronic devices that detect different physical values around/on them. Traditional IoT systems usually require users to explicitly set the sensors that define their status; there are other cases where the devices have the cognitive ability to process the data directly and understand the changing status of the user. In both cases, the knowledge is stored and processed in a central platform. The VU exploits a logic in this scenario to know which VOs abstract the device features that describe the user’s state and the surrounding environment. Through this virtualization-level exchange of data, learning mechanisms provide a dynamic portion of the user profile describing behaviors and preferences. In this way, the users are relieved from this duty, and the knowledge is stored and processed for each user in the related VU, ensuring more flexibility and a better perception of privacy. Indeed, the VU runs in the cloud space of the user, and the cloud provider manages the user’s data storage space without having knowledge of the contents that remain at the exclusive availability of the user. The VU interacts directly with the VOs of the objects owned by the same person represented by the VU in all these processes.

In order to simplify the ID&A management, instead of requiring the user to set the credentials as it is usually done in traditional IoT systems, the VU generates keys for accessing its resources, diversifying them depending on either the output or the node that requests the resource. In this way, the ID&A manager, when called by services or applications of other users, can check the access privileges of the requesting user. Information about the user context can be added on the fly to the semantic description of requested resources and to be aggregated, to reduce latency in service discovery and to improve the accuracy of results.

Given the complexity of providing details about the implementation of all the VU functionalities described above, as a first step in this paper, we focus on the implementation of the core VU functionality, i.e., the creation and dynamic update of the VU profile based on users’ context, which is conducted on the basis of data gathered by their VOs. Indeed, as mentioned above, VU profiles are created and updated according to data collected by ICT objects. In the following steps, we describe a simple mechanism to create a VU profile based on context-specific data. In order for the VU profile to be enriched with information about user preferences (e.g., in terms of temperature and light), each time the user enters a new location the VOs that describe the ICT objects of that room are discovered, and new relationships are created. Accordingly, once relationships are established between the VOs and the VU, the data gathered by VOs can be used by the VU to update its profile. Since preferences may be different for each location visited by the user, data need to be properly processed, by means of a clustering algorithm, so that different preferences are associated with different locations. Specifically, the VU acts as follows:

1. The VU acquires user’s data H24 with a given sampling frequency;
2. Once a sufficient amount of data has been collected, the VU computes the portions of the profile related to the clusters;
3. The VU detects the preferred settings related to the clusters above and stores them into the DNA;
4. For each newly acquired sample, the classifier detects the relevant preferences;
5. The VU sends to the user’s VOs the settings to be applied;
6. At the end of the day, new parts of the profile are computed based on data values gathered during the day. The preferred setting values are updated accordingly.

Daily updates allow the system to easily adapt to ambient changes, e.g., to seasonal changes in external temperature, so that the most appropriate preferred values are always chosen.

As a reference example, we suppose to monitor the temperature and that two preferences, *preference1* and *preference2*, have been detected: the average comfort value for *preference1* is the average temperature value ${\bar{T}}_{1}$, while the average comfort value for *preference2* is the average temperature value ${\bar{T}}_{2}$. These values are stored inside the user model, along with the other data related to the user profile. As it will be better explained in the following, users’ preferences will then be used to set the appropriate settings (computed by taking into account all the acquired features for the considered profile) to other locations where users are associated with other VOs. When new data are acquired, each group of samples collected by the VU is classified by means of a model obtained by training a classifier. As a result, samples are assigned to one of the preferences detected by the clustering algorithm. Based on it, the VU sends to the appropriate VO, located in the same location as the user, user-related settings, so that they can adapt according to the user’s needs.

## 5. Legal Aspects Related to the Virtual User

### 5.1. Legal Issues

The introduction of a virtual “alter ego” of users, namely the VU, raises other interesting considerations from a legal point of view. The VU is autonomous from any human activity, reacts to external stimuli and is suitable for possible changes. At the same time, it maintains its identity. In fact, it is able to interact with other agents, yet it differs from them.

In this scenario, it is of fundamental importance to understand *who* and in *what capacity* should be accused of the legal effects of VU activities. This problem arises because they are not legal entities for the legal system and, consequently, there cannot be right holders. Usually, right holders are human beings, but, in most modern jurisdictions, bodies (companies, hospitals, associations) and, sometimes, animals can also be holders of rights. The laws of legal systems all over the world contemplate software as an object of copyright, so the owner of the right is still the human being. Legal doctrine has often asked who should be held responsible for the damages caused by the software, reasoning about the possibility of ascribing them to the producer (programmer) or to the user. However, these topics have always been about inanimate software. In the case of the VU, the scenario is very different, as it is able to *understand reality*, *build its own experience* and *decide how to behave* based on what it has learned.

### 5.2. Current Status of Legislation

To address this issue, the literature about rights and responsibilities of robots is of great help [24,25,26]. Most of these studies consider robots as beings composed of a mechanical part (hardware) and a brain part (software). A VU lacks a tangible part, which is totally replaced by the software part. Nevertheless, most of the studies developed on legal aspects of robotics can also be used for virtual machines. This is the case for instance of the autonomous driving car [27], where everything revolves around the software part because on the hardware side the legal systems are widely developed. Thus, self-driving cars, drones, robotic surgery systems, domestic robots (all of which are also equipped with a hardware part) are not different from virtual personal assistants (composed only of software).

Currently, there is no specific legislative measure to govern subjects such as the VU, but, given the previous analogy, the recommendation of the European Union in [28] can be used as a reference. Indeed, most of the considerations contained in it can also refer to software agents (without the hardware part) because they are aimed at the mental aspect, i.e., agents capable of performing activities that were traditionally and exclusively human, which have developed certain autonomous and cognitive characteristics (ability to learn from experience and make decisions almost independently) that allow them to interact with the surrounding environment and to alter it significantly (see Z clause in [28]). In this context, as the Virtual User can take autonomous decisions, traditional standards are not sufficient to activate the liability for the caused damages caused by them *as they would not allow for determining which is the responsibility of the compensation nor to demand from that subject the repair of the damages caused* (see AF clause in [28]).

### 5.3. Future Perspectives

The EU Resolution clearly expresses the awareness that linking the activity of machines to that of a single human manager will be increasingly difficult. Therefore, an independent legal entity should be chosen to achieve the creation of the “electronic person” category, who is responsible to indemnify any damage caused by it. The creation of this new category must certainly not be limited to civil liability for damages but to every aspect relevant to its management. Consider, for example, criminal liability, which is based on the capacity of understanding and on the will of the agent. This can lead someone to wonder if the VU can have its own capacity to understand and to decide.

The possibility of creating an electronic legal personality is viewed with disfavor by the European Economic and Social Committee, which, for reasons mainly of ethical nature, expressed its opposition in the opinion INT/086 of 31 May 2017. Nevertheless, the EU resolution posed a series of ethical precautions to preserve human dignity and made also express reference to the protection of personal data. This aspect has been addressed by a legal doctrine since the beginning of the 1990s, when the problem of the relationship between artificial intelligence and fundamental human rights began to be addressed, arguing that development had to move in respect of human rights and not towards the “Great Brother’s rule” [29,30]. The risk is very high because the characteristics of each human person could be concentrated in large databases, from which information, even very confidential, about the lives of people can be obtained to be used for illicit purposes. The rules to protect personal data from unlawful use are today contained in the EU Reg. 2016/679, General Data Protection Regulation (GDPR), which is characterized by the attention that is placed on the accountability of data controllers’ and processors’ data.

The system outlined by European legislators is based on the assessment of risks (for the rights and freedoms of natural persons) deriving from the specific activity of processing personal data. All the concrete measures that will be put in place on the basis of this evaluation are handed back directly to the data controller. The data controller must always minimize the processing of personal data, give maximum transparency on the purposes and methods of processing personal data, allow the data subject to control the processing by making the rights provided by the Regulation easily and effectively excitable [31,32,33,34,35,36,37]. The data controller must comply with these criteria in all the processing phases: in the development, design, collection, selection and use of personal data, and always in the light of a careful analysis of the specific reference context.

In the same way, the “data protection by design and by default” principle must be respected by the VU presented in this paper. A preliminary assessment is needed on the type of data processed and on the data necessary to pursue the processing purpose. Once these initial assessments have been made, minimization will have to be applied. Accordingly, unnecessary data has to be deleted, data for which it is not necessary to maintain a connection with the identity of people have to be anonymized, and information that might be necessary to reconnect to the people concerned have to be pseudonymized. Finally, data controllers have to assess risks and apply appropriate measures to limit them. In the end, it is important to stress that, in an application scenario such as the one analyzed in this paper, constant monitoring is needed because the information processing performed by the VU may have unexpected scenarios following the application of additional features or following the autonomous evolution of the software agent.

## 6. Performance Evaluation

Once the VU has been able to create an accurate profile of the user, this is used for setting preferences in the applications with the user interface in such a way that a better QoE is provided. With reference to this VU functionality and application scenario, in the following, we describe the specific use case we focus on.

In particular, for this evaluation, we consider the following data: position, temperature and ambient light, collected from sensors inside users’ personal smartphones.

In the following subsection, i.e., Section 6.1, the logic of the reference use case used to evaluate the performance of the VU-enabled system will be explained. In Section 6.2, the VU-enabled profiling is compared with manual profiling carried out by means of a smartphone app. Finally, in Section 6.3, the latency and computational load required to create and manage VU-enabled profiles are analyzed and compared to the case of VO-enabled profiling.

### 6.1. Use Case Logic

To detect the correct user preferences, an unsupervised machine learning algorithm with k-means clustering has been chosen [38]. More specifically, we used the GAP statistic method defined in [39]. This method is based on the definition of Gap(k) with respect to the number of clusters *k*

(1) $$Gap\left(k\right)=E^{*}log(W_{k})-log\left(W_{k}\right),$$

where $W_{k}$ is the inertia with respect to *k*, i.e., the sum of the pairwise distances of the intra-cluster data, and $E^{*}$ is the expected value for the null reference distribution of the data, i.e., a distribution where there is no underlying clustering. The reference distribution we considered is a uniform distribution. The number of clusters is chosen as the lowest value of *k* that satisfies the inequality

(2) $$Gap\left(k\right)\geq Gap\left(k+1\right)-s_{k+1},$$

where $s_{k+1}$ is the standard error of the distribution for a number k+1 of clusters.

The following subsection describes how the GAP statistic method can be used to define the number of different preferences that define the VU profile. Accordingly, a set of data collected from several sensors belonging to the same smartphone is analyzed to compute the optimal number of clusters that can be used to profile a user. Subsequently, the effectiveness of the automatic profiling performed by the VU (i.e., the VU-enabled profiling) using this dataset is evaluated by comparing it with a manually-set profile that is used to control an air conditioning system. Note that the GAP statistic method is used in this evaluation section just for demonstration purposes: other more complex and more efficient machine learning algorithms can be used to get more accurate results. Our objective here is to prove that good performance of the VU-enabled system can be obtained also using simple mechanisms such as the one described.

### 6.2. Performance of the Profiling Function

**Figure 6:**
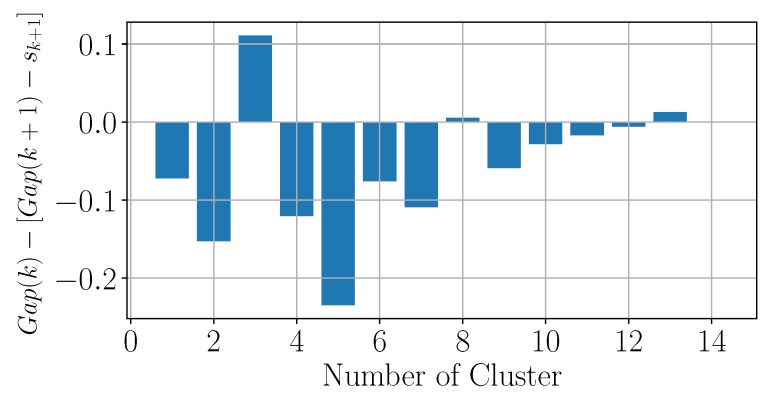
Evaluation of the optimal number of clusters using the GAP statistic method.

In order to evaluate the performance of the VU, we first focus on identifying the reasonable number of clusters that ensures a good model. Simulations have been carried out using 1600 samples with 16 features each, and a sampling frequency of one sample every 20 min. Based on the acquired data, the number of clusters computed according to Equation (2) is k=3, as shown in Figure 6.

Based on this result, we then compare the automatic profiling performed by the VU, namely the VU-enabled profiling, with an already known manually-set profile. More specifically, we compare the preferences that result from the VU-enabled profiling with those that are set by the user by means of a smartphone app. In order to test how the VU-enabled profiling works under borderline conditions, test settings are chosen so that the criteria that are used to perform the VU-enabled profiling, i.e., monitored temperature values and user locations are very similar to each other.

**Figure 7:**
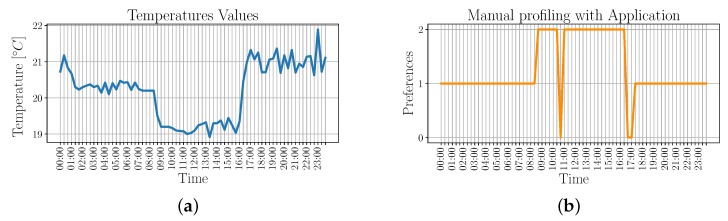
Temperature values and identified profiles for the manually-set profile. Note that each profile corresponds to a different location. (**a**) Temperature values w.r.t. time; (**b**) Identified profile w.r.t. time.

We first used manual settings to set the air conditioning system of two specific locations based on the user preferred temperature. The first location, i.e., home with coordinates lat:39.23538, lon:9.10322, is associated with *preference1* and corresponds to a preferred temperature value of ${\bar{T}}_{1}=$$21\;{}^{{}^{\circ}}C$, with a tolerance of ±1 ${}^{{}^{\circ}}$C. The second location, i.e., workplace with coordinates lat:39.22932, lon:9.10992, is associated with *preference2* and corresponds to a preferred temperature of ${\bar{T}}_{2}=$$19\;{}^{{}^{\circ}}C$ with a tolerance of ±1 ${}^{{}^{\circ}}$C. When the user is located outside both locations, the selected preference is *preference0*, which is not associated with any preferred temperature. Figure 7 shows the temperature values that are monitored by a temperature sensor in the different locations, along with the corresponding preferences.

**Figure 8:**
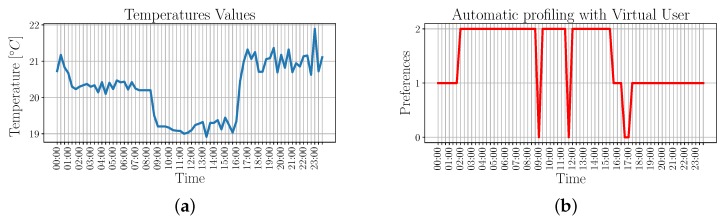
Temperature values and identified profiles for the VU-enabled automatic profiling. (**a**) Temperature values w.r.t. time; (**b**) Identified profile w.r.t. time.

During VU-enabled profiling training, user’s preferred temperatures are not known in advance, but they are rather derived from the acquisitions made during the day so that they can be later used to appropriately control the air conditioning system. These acquisitions are based on the monitored temperature and the user’s position. Note that both monitored temperature and user’s position are very close to each other. The resulting preference IDs with respect to the time of the day are shown in Figure 8.

By comparing the results of Figure 7 and Figure 8b, it can be noticed that the identified behaviors correspond, with the exception of the period between midnight and 8:40 a.m., when the VU-enabled profiling associates the acquired data to *preference2* instead of *preference1*. This behavior is due to the high similarity with the acquisitions made between 9:00 a.m. and 3:40 p.m., such that they are classified as belonging to the same cluster.

Nevertheless, as it is proven by Table 1, the average temperature monitored using the two classification results are very close to each other, and also very close to the user’s preferred temperature.

**Figure 9:**
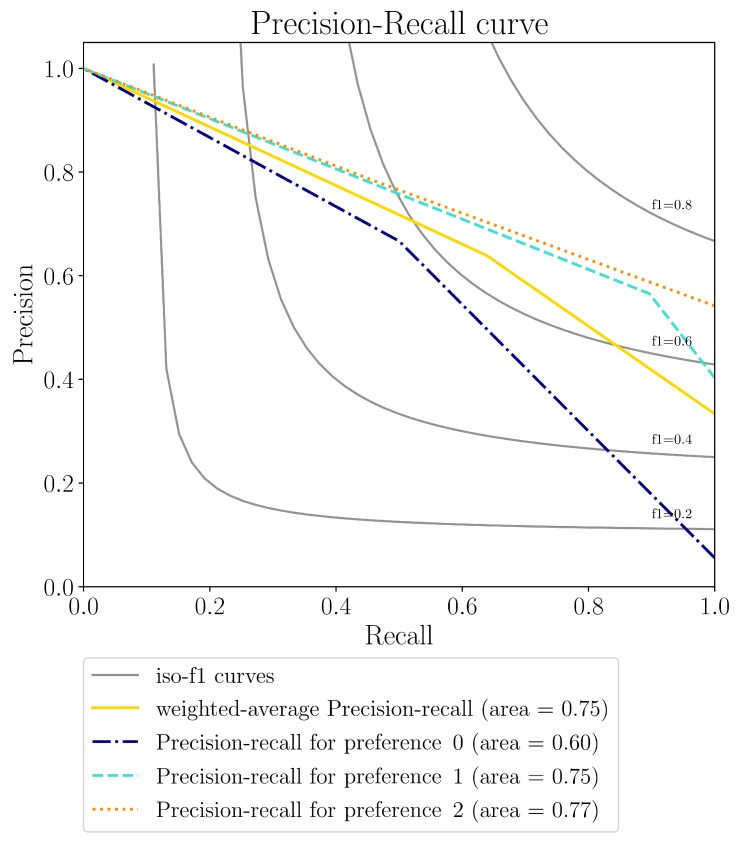
Precision–Recall curve for the VU-enabled profiling w.r.t. manual profiling.

Figure 9 shows the precision–recall curve of the VU-enabled profiling with respect to the manually-set profile. Note that a high precision corresponds to a low number of false positives, while a high recall corresponds to a low number of false negatives. Results are quite good for both *preference1* and *preference2*, but not very much for *preference0*. The reason is that the number of samples for *preference0* is considerably lower than that of the other two preferences, and thus the impact of an error is very high. This is also proved by the weighted-average precision–recall, where the weight is given by the number of samples.

### 6.3. Latency and Computational Load Evaluation

In order to evaluate the impact of latency and computational load required by profile creation and management in the VU-enabled system, we compare it with a VO-enabled system where a profile is created and associated with each VO. Simulations are performed considering three VOs running on the Lysis platform [19,40], equipped with different numbers of sensors, as reported in Table 2. Lysis platform is an implementation of the Social Internet of Things paradigm (SIoT). The VOs in SIoT have the capacity to socialize and establish relationships based on their owners’ behaviour. Therefore, the interactions between objects, users, and the contexts translate into distinct kinds of relationships. The VOs used in the experiment exploit interactions of co-location at home and in the office and ownership relationships to make the VU understand which VOs to observe in order to obtain the profile of the user in those situations.

In the VO-enabled system, each VO has to autonomously create a profile associated with it. To this, it first requests to the other VOs belonging to the same user the preferences related to each of their sensors. Analogously, it replies to each VO with the preferences associated with its own sensors. Therefore, with this profiling scheme, the number of requests and answers are as detailed in Table 2.

In the VU-enabled profiling, the VU is in charge of the profiling process, and it collects the data needed to create the profile. The VOs do not have to request data to the other VOs, thus offloading the computational load. The VU sends a request and receives a reply for each of the involved sensors, as presented also in Table 2.

**Figure 10:**
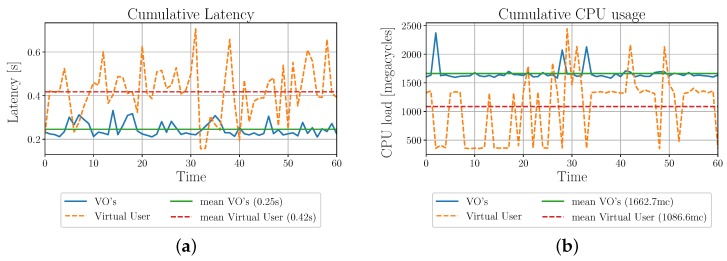
Comparison of latency and computational load for the VO-enabled and VU-enabled profiling. (**a**) Latency w.r.t. time; (**b**) CPU computational load w.r.t. time.

To test the latency and computational load of the described systems, tests were performed running the profiling process every minute for an hour, for a total of 60 profiling cycles for both cases. The performance of the two systems is shown in Figure 10 in terms of average latency and CPU megacycles.

As shown in Figure 10b, latency is around 40% higher for the VU-enabled profiling with respect to the VO-enabled profiling. This is due to the fact that the number of requests that the VU has to perform is higher, thus leading to higher waiting times. Nevertheless, the resource consumption for the VU-enabled profiling is around 50% lower than that of the VO-enabled profiling. This is mostly due to the lower load of VOs in the latter case, which drastically lowers the number of requests to be satisfied. Therefore, VU-enabled profiling is particularly preferable for applications that do not have real-time requirements, but where resource saving is critical.

## 7. Conclusions

This paper introduces the concept of Virtual User, which is intended as the virtual counterpart of the user and implemented as an agent running in the cloud/edge computing infrastructure able to pretend to be the user in the process of IoT service providing. The VU is realized through key components, such as those that deal with: profiling, owner control, social network management, ID&A management, QoE management, migration management, and context management.

From the analysis of the needed functionalities, it appears that the user preferences and the IoT context predictions are the most important ones. In our experiments, we have evaluated the capability of the VU to identify the profile of the user during the day, which changes depending on the context. The identification of the profile has then been used to set the air conditioning system. The results have shown that, for two profiles out of three assigned to the user, the system is able to identify the correct one automatically with a precision–recall area equal or higher than 0.75. For the other profile, the precision–recall area is of 0.60. As an effect, the system has been able to set the correct temperature for most of the time, i.e., close to the one that would have been set manually by the user. In fact, the difference between the set temperature and the desired setting is as low as 0.37 Celsius degrees. Still, there is space for improvements. The low number of observations of the user interactions is one of the weaknesses; indeed, increasing this number, it would be easy to reach better performance allowing for a better training of the classifier. The observations could also be improved by adding the number of sensors to identify the context of the user. For instance, some external sensors of luminosity other than the one on the user terminal would allow for a more precise measurement of the light intensity, which is now affected by the position of the terminal. One of the major activities we are working on for future improvement is indeed the introduction of additional sensors.

Another important result that is highlighted in the conclusions is that the introduction of the VU allows for a reduction in the usage of resources. Indeed, when the VU is not used, each VO has to autonomously create a profile associated with it, which intensifies the communications among the VOs. Differently, in the VU-enabled profiling, the VU is in charge of the profiling process, and it collects the data needed to create the profile. The VOs do not have to request data to the other VOs, thus offloading the computational load. As a result, in the considered scenario, the resource consumption for the VU-enabled profiling is around 50% lower than that of the case when the VU is not used, at the expenses of a little increase in the communications latency. Therefore, VU-enabled profiling is particularly preferable for applications that do not have real-time requirements, but where resource saving is critical.

## Figures and Tables

**Table 1:** Comparison of the average values of temperature for the VU-enabled profiling and for the manually-set profile, and the preferred temperature, for each identified preference.

Preference ID	${\bar{T}}_{\mathrm{VU}}{[}^{{}^{\circ}}C]$	${\bar{T}}_{\mathrm{Man}}{[}^{{}^{\circ}}C]$	$T_{\mathrm{Pref}}{[}^{{}^{\circ}}C]$
Preference0	20.139	20.401	——
Preference1	20.821	20.660	21
Preference2	19.767	19.398	19

**Table 2:** Comparison of requests and answers to create and manage profiles using the VO-enabled and VU-enabled profiling.

Name	# of Sensors	VO-Enabled		VU-Enabled	
		# of req.	# of ans.	# of req.	# of ans.
VO1	2	15	4	0	2
VO2	10	7	20	0	10
VO3	5	12	10	0	5
VU	NA	NA	NA	17	0
Tot.	17	34	34	17	17
